# MicroRNA Mediated Changes in Drug Metabolism and Target Gene Expression by Efavirenz and Rifampicin *In Vitro*: Clinical Implications

**DOI:** 10.1089/omi.2019.0122

**Published:** 2019-10-04

**Authors:** Marelize Swart, Collet Dandara

**Affiliations:** ^1^ Division of Human Genetics, Department of Pathology, University of Cape Town, Cape Town, South Africa.; ^2^ Institute of Infectious Disease and Molecular Medicine, Faculty of Health Sciences, University of Cape Town, Cape Town, South Africa.

**Keywords:** drug metabolism, efavirenz, rifampicin, HepaRG cells, microRNA, *CYP2B6*, *CYP3A4*

## Abstract

Efavirenz (EFV) and rifampicin (RMP) are widely prescribed in Africa for treatment of HIV/AIDS and tuberculosis epidemics. Exposure to medicines can alter drug metabolism, for example, through changes in expression of microRNAs. We report, in this study, novel observations on the ways in which EFV and RMP change microRNA expression signatures *in vitro* in HepaRG cells. Additionally, we discuss the clinical implications of changes in expression of drug-metabolizing enzyme genes, such as *CYP3A4*, *CYP3A5*, *UGT1A1*, *CYP2B6*, and *NR1I3*. Differentiated HepaRG cells were treated with EFV (6.4 μM) or RMP (24.4 μM) for 24 h. Treatment of HepaRG cells with EFV resulted in a significant increase in messenger RNA (mRNA) expression for *CYP3A4* (12.51-fold, *p* = 0.002), *CYP3A5* (2.10-fold, *p* = 0.019), and *UGT1A1* (2.52-fold, *p* = 0.005), whereas *NR1I3* expression decreased (0.41-fold, *p* = 0.02). On the other hand, treatment of HepaRG cells with RMP resulted in a significant increase in mRNA expression for *CYP2B6* (6.68-fold, *p* = 0.007) and *CYP3A4* (111.96-fold, *p* = 0.001), whereas *NR1I3* expression decreased (0.46-fold, *p* = 0.033). These data point to several important clinical implications through changes in drug/drug interaction risks and achieving optimal therapeutics. All in all, this study shows that differential expression of microRNAs after treatment with EFV and RMP adds another layer of complexity that should be incorporated in pharmacogenomic algorithms to render drug response more predictable.

## Introduction

Efavirenz (EFV) is widely used in combination antiretroviral therapy to treat HIV/AIDS, whereas rifampicin (RMP) is a component of standardized antituberculosis treatment. Pharmacokinetics and pharmacodynamics of EFV and RMP are affected by many factors, including age, body weight, gene variations within genes coding for enzymes involved in drug disposition, and microRNAs (Zanger et al., 2018).

MicroRNAs are noncoding, endogenous, single-stranded RNA molecules and often 21–23 nucleotides in size. More than 2500 mature human microRNAs have been identified (Kozomara et al., 2019). These microRNAs are thought to play a major role in posttranscriptional regulation of messenger RNAs (mRNAs) (Friedman et al., 2009), including genes coding for enzymes involved in drug disposition.

In one of the first studies illustrating the role microRNAs play in drug metabolism, Takagi et al. (2008) showed that miR-148a is involved in posttranscriptional regulation of *NR1I2* (pregnane X receptor [PXR]) mRNA expression by binding to its 3′-UTR (untranslated region). Since then, microRNAs have been shown to play an important role in regulation of many genes coding for drug-metabolizing enzymes (DMEs), drug transporters, and nuclear receptors (NRs) (Dluzen and Lazarus, 2015; Dreussi et al., 2012; Glubb and Innocenti, 2011; Gomez and Ingelman-Sundberg, 2009a, 2009b; Haenisch and Cascorbi, 2012; Ikemura et al., 2014; Ingelman-Sundberg et al., 2007; Klaassen et al., 2011; Nakajima and Yokoi, 2011; Rieger et al., 2013; Rukov and Shomron, 2011; Singh et al., 2011; Yokoi and Nakajima, 2013; Yu, 2007, 2009; Zhang and Dolan, 2010), such as *CYP3A4* (Pan et al., 2009) and *NR1I2* (Takagi et al., 2008).

The present study reports novel observations on the ways in which EFV and RMP change microRNA expression signatures *in vitro* in HepaRG cells. Additionally, we discuss the clinical implications of changes in expression of DME genes, such as *CYP2B6*, *CYP3A4*, *CYP3A5*, *UGT1A1*, and *NR1I3*.

## Materials and Methods

### HepaRG cell culture

Differentiated HepaRG cells have been derived from a hepatocellular carcinoma cell line and were obtained from Merck Millipore (EMD Millipore, Billerica, MA, USA). Differentiated HepaRG cells were cultured as an adherent cell line (70% confluency) using collagen I-coated 24-well plates (Thermo Fisher Scientific, Waltham, MA, USA). Base medium contained William's Medium E (Thermo Fisher Scientific) and 1% GlutaMAX^™^ (Thermo Fisher Scientific). HepaRG thawing/plating medium was prepared by adding 12.5 mL HepaRG thawing/plating medium supplement (Thermo Fisher Scientific) to 100 mL base medium. HepaRG culture medium was prepared by adding 14 mL HepaRG culture medium supplement (Thermo Fisher Scientific) to 100 mL base medium. HepaRG serum-free induction medium was prepared by adding 0.6 mL HepaRG serum-free induction medium supplement (Thermo Fisher Scientific) to 100 mL base medium. Culture conditions were 37°C with 5% CO_2_ and 95% relative humidity.

### Treatment of HepaRG cells with EFV or RMP

EFV and RMP (Sigma-Aldrich, St Louis, MO, USA) were dissolved in 100% dimethyl sulfoxide (DMSO; Sigma-Aldrich) to a stock concentration of 32 and 122 mM, respectively. HepaRG serum-free induction medium was used to dilute EFV and RMP before treatment of HepaRG cells. After thawing and seeding, differentiated HepaRG cells were maintained in HepaRG culture medium (renewed on days 4 and 6 after cell thawing) for 7 days. HepaRG culture medium was replaced with HepaRG serum-free induction medium containing EFV at a concentration of 6.4 μM (0.02% DMSO for the solvent-treated control) and RMP at a concentration of 24.4 μM (0.02% DMSO for the solvent-treated control).

Clinically relevant plasma concentrations among HIV/AIDS or tuberculosis patients are 1–4 μg/mL (3.2–12.7 μM) for EFV and 8–24 μg/mL (9.7–29.2 μM) for RMP. Hence, the drug concentrations were chosen with guidance by this information to maximize the clinical relevance of the present study. Treatments were carried out for 24 h and three biological replicates were available for each of the different treatment conditions (EFV, RMP, and DMSO).

### RNA isolation

Total RNA was isolated from HepaRG cells, after treatment, using the Quick-RNA^™^ MiniPrep Kit (Zymo Research Corporation, Irvine, CA, USA) as per the manufacturer's instructions. Briefly, serum-free medium containing DMSO or EFV/RMP was aspirated before washing the cell layer twice with ice-cold 1 × phosphate-buffered saline. Thereafter, 600 μL RNA lysis buffer was added to each well and followed by pipette mixing before transferring samples to a 1.5-mL Eppendorf tube (DEPC treated). Cell debris was removed by centrifugation at 14,000 rpm for 1 min before each sample was transferred to a Spin-Away column. Genomic DNA was removed by centrifugation at 14,000 rpm for 1 min. Six hundred microliters 100% ETOH (DEPC treated) was used to precipitate RNA and each sample was transferred to a Zymo-Spin IIICG column.

The column was washed to remove 100% ETOH before in-column DNase I treatment and washed again with RNA prep buffer and RNA wash buffer before RNA elution. Thirty microliters of DNase/RNase-free water (prewarmed to 95°C) was used for RNA elution into a RNase-free 1.5-mL Eppendorf tube and this step was repeated. All RNA samples were quantified using both a NanoDrop^®^ ND-1000 Spectrophotometer (Thermo Fisher Scientific) and the Agilent^®^ RNA 6000 Nano Kit on an “Agilent 2100 Bioanalyzer Instrument” (Agilent Technologies, Inc., Santa Clara, CA, USA).

### Complementary DNA synthesis and mRNA expression profiling by quantitative polymerase chain reaction

The Maxima H Minus First-Strand Complementary DNA (cDNA) Synthesis Kit (Thermo Fisher Scientific) was used according to the manufacturer's instructions. The cDNA synthesis reaction components included 1 μg total RNA, 0.31 pmol oligo(dT)_18_ primer, 0.31 pmol random hexamer primer, 0.5 mM dNTPs, and nuclease-free sdH_2_O up to a volume of 15 μL. Components were added to a sterile nuclease-free tube on ice and incubated at 65°C for 5 min. Samples were cooled on ice for a further 5 min before adding the 5 × RT (reverse transcriptase) buffer and RevertAid^™^ Premium Enzyme Mix. Samples were mixed and incubated for 10 min at 25°C, followed by 15 min at 50°C and 5 min at 85°C on the “T100 Thermal Cycler” from Bio-Rad Laboratories, Inc. (Hercules, CA, USA). cDNA was stored at −20°C until it was used for quantitative polymerase chain reaction (qPCR).

Hydroxymethylbilane synthase (*HMBS*), TATA-binding protein (*TBP*), and succinate dehydrogenase complex, subunit A, flavoprotein (*SDHA*) genes were assessed as reference genes for normalization during qPCR based on findings by Ceelen et al. (2011) in HepaRG cells. Table 1 shows qPCR conditions, primer sequences, and qPCR amplification product sizes. NCBI Primer-BLAST (Altschul et al., 1990), which can be found at the following URL: www.ncbi.nlm.nih.gov/tools/primer-blast/, was used to determine the specificity of qPCR primers. Furthermore, dissociation curve analysis, agarose gel electrophoresis, and direct cycle sequencing was used to verify specificity of qPCR primers.

**Table 1.** Quantitative Polymerase Chain Reaction Amplification Conditions Used for Messenger RNA Expression Profiling

*Gene*	*Primer sequence (5′-3′)*	*Annealing temperature (°C)*	*qPCR amplification product size (bp)*	*References*
CYP1A2	F: TCGTAAACCAGTGGCAGGT; R: GGTCAGGTCGACTTTCACG	64	254	Wilkening and Bader (2003), Wilkening et al. (2003)
CYP2B6	F: TTCCTACTGCTTCCGTCTATCAAA; R: GTGCAGAATCCCACAGCTCA	62	67	Antherieu et al. (2010)
CYP3A4	F: CTTCATCCAATGGACTGCATAAAT; R: TCCCAAGTATAACACTCTACACAGACAA	62	87	Antherieu et al. (2010)
CYP3A5	F: TGACCCAAAGTACTGGACAG; R: TGAAGAAGTCCTTGCGTGTC	65	240	Rodriguez-Antona et al. (2001)
UGT1A1	F: TGACGCCTCGTTGTACATCAG; R: CCTCCCTTTGGAATGGCAC	62	74	Antherieu et al. (2010)
UGT2B7	F: GGAGAATTTCATCATGCAACAGA; R: CAGAACTTTCTAGTTATGTCACCAAATATTG	62	123	Ohno and Nakajin (2009)
SULT1A1	F: AACGCAAAGGATGTGGCA; R: TCCGTAGGACACTTCTCCGA	62	120	Miyano et al. (2005)
NR1I2	F: CCAGGACATACACCCCTTTG; R: CTACCTGTGATGCCGAACAA	62	60	Antherieu et al. (2010)
NR1I3	F: TGATCAGCTGCAAGAGGAGA; R: AGGCCTAGCAACTTCGCATA	62	102	Antherieu et al. (2010)
TBP	F: GAGAGTTCTGGGATTGTACCG; R: ATCCTCATGATTACCGCAGC	62	143	Ceelen et al. (2011)
HMBS	F: CTGTTTACCAAGGAGCTTGAAC; R: TGAAGCCAGGAGGAAGCA	62	100	Ceelen et al. (2011)
SDHA	F: CGGCATTCCCACCAACTACA; R: GCTGATTTTCCCACAACCTTC	65	388	Ceelen et al. (2011)

HMBS, hydroxymethylbilane synthase; qPCR, quantitative polymerase chain reaction; SDHA, succinate dehydrogenase complex, subunit A, flavoprotein; TBP, TATA-binding protein.

A “CFX96 Thermal Cycler” from Bio-Rad Laboratories, Inc. and white 96-well PCR plates (Starlab Ltd., Milton Keynes, United Kingdom) were used to perform qPCR reactions. qPCR conditions were 95°C for 3 min, followed by 40 cycles of 95°C for 10 sec, the specific annealing temperature of each primer set (Table 1) for 20 sec, and finally the dissociation curve of 65°C to 95°C in 0.5°C increments. The SYBR FAST Universal qPCR Kit from KAPA Biosystems (Kapa Biosystems, Inc., Wilmington, MA, USA) was used and each reaction was performed in triplicate for each of the three biological replicates of the treatment conditions.

Each qPCR reaction contained the following components: 10 ng cDNA, 0.2 μM of each primer, 1 × SYBR FAST MIX, and was made up to a total volume of 10 μL with sdH_2_O. A no-template control and no-RT control (sample that did not undergo reverse transcription or cDNA synthesis) was included for each primer set to ensure no contamination.

### Statistical analyses for differential mRNA expression

The relative standard curve analysis method was used to analyze qPCR data by performing a standard curve for each primer set (cDNA concentrations of 25, 12.5, 6.25, and 3.125 ng) (Pfaffl, 2001). The following values were calculated based on the standard curve: *r*^2^, % amplification efficiency, slope of curve, and *y*-intercept. The obtained Cq values of each test and reference gene, for each technical replicate, were plotted onto the standard curve of each primer set. The Cq values and standard curve, for each primer set, were used to calculate the log input value [*x* = (Cq-*y*-intercept)/slope) for each technical replicate. The log input value was then used to calculate the input value (10:log input value). The average input value and standard error were calculated between technical replicates for each test or reference gene.

The ratio of test/reference gene average input values between technical replicates were then calculated to determine if a test gene is up- or downregulated compared with the reference gene. Finally, the fold change was calculated by using the normalized ratio of input values to calculate the ratio of treated/solvent-treated samples for each biological replicate. To determine which mRNAs are differentially expressed for EFV- versus DMSO-treated cells or RMP- versus DMSO-treated cells, *t*-test was used, across technical and biological replicates, to assess the statistical significance of changes in mRNA expression (*p* < 0.05).

### Statistical analyses for microRNA differential expression

MicroRNA expression profiling for 754 microRNAs was performed using the TaqMan^®^ OpenArray^®^ Human MicroRNA Panel and QuantStudio^™^ 12K Flex system (Thermo Fisher Scientific) according to the manufacturer's protocol. Data were analyzed with the R/Bioconductor package “Automated Analysis of High-Throughput qPCR Data” (Dvinge and Bertone, 2009). C_T_ values were used for differential expression analysis and microRNAs with a C_T_ value >35 was considered undetected. The endogenous controls (RNU44, RNU48, U6, and Ath-miR159a) were excluded from further analysis. MicroRNAs undetected in any of the replicate samples or microRNAs with AmpScore <1.24 or CqConf <0.8 were excluded.

Quantile normalization was followed by limma analyses to identify differentially expressed microRNAs for EFV versus DMSO and RMP versus DMSO (with the R/Bioconductor package “Automated Analysis of High-Throughput qPCR Data”). Quantile normalization is used to minimize variability between TaqMan OpenArray Human MicroRNA Panels and assumes that most microRNAs are not differentially expressed. Limma analysis involves fitting of a one-factorial linear model for each microRNA between EFV- or RMP-treated and DMSO-treated replicates and the standard errors are moderated using an empirical Bayes model resulting in moderated *t*-statistics for each microRNA (Diboun et al., 2006; Ritchie et al., 2015). *p*-Values <0.05 were considered statistically significant and adjusted *p*-values are reported after correction for multiple testing using the Benjamini/Holm method.

### MicroRNA target gene identification

Potential target mRNAs for each of the differentially expressed microRNAs was identified by using the bioinformatic prediction algorithm Ingenuity Pathway Analysis (IPA), and by searching MiRTarBase database with known microRNA/mRNA interactions or microRNA/mRNA pairs with negatively correlated expression. Target prediction was performed with the IPA microRNA/target prediction algorithm. MiRTarBase was used to search for experimentally validated microRNA/target interactions. Target genes obtained from IPA and MiRTarBase were filtered for 300 genes of pharmacokinetic and pharmacodynamic relevance.

## Results

### Effects of EFV or RMP on HepaRG cell morphology

HepaRG cells were treated with 6.4 μM EFV (0.02% DMSO for the solvent-treated control) or 24.4 μM RMP (0.02% DMSO for the solvent-treated control) for 24 h. HepaRG cell morphology was observed and photographed before and after treatment with DMSO, EFV, and RMP. Treatment with EFV, RMP, or DMSO did not alter cell morphology (Supplementary Fig. S1A–C).

### Effects of EFV or RMP on mRNA expression

Individual effects of EFV or RMP treatment on mRNA expression of *CYP1A2*, *CYP2B6*, *CYP3A4*, *CYP3A5*, *UGT1A1*, *UGT2B7*, *SULT1A1*, *NR1I2*, and *NR1I3* were evaluated *in vitro* in HepaRG cells. Changes in mRNA expression were measured after treatment with EFV (6.4 μM) and RMP (24.4 μM) for 24 h. qPCR results are represented as fold changes in mRNA expression relative to the control samples (treated with 0.02% DMSO). A fold expression of one represents expression at the same level as that of the control samples (DMSO treated). A change in mRNA expression above one shows an increase in expression, whereas a change in expression below one indicates a decrease in expression, relative to the DMSO-treated control samples.

**FIG. 1.**
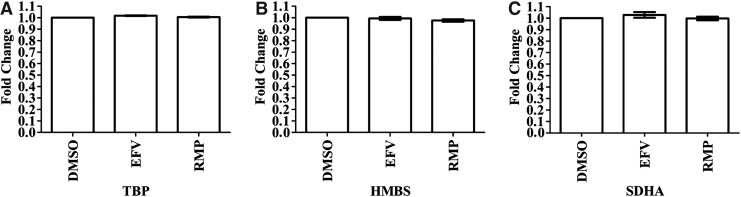
Comparison of mRNA expression between the three treatment conditions (efavirenz, rifampicin, and DMSO) showed no effect of efavirenz and rifampicin on mRNA expression for any of the three reference genes. Differentiated HepaRG cells were treated with efavirenz (6.4 μM), rifampicin (24.4 μM), or DMSO (0.02%) for 24 h (including three biological replicates). Expression of mRNA for three reference genes, in triplicate, were assessed by using qPCR and the fold change in mRNA expression is compared for each treatment condition and for each reference gene. **(A)** TBP; **(B)** HMBS; **(C)** SDHA. DMSO, dimethyl sulfoxide; HMBS, hydroxymethylbilane synthase; mRNA, messenger RNA; qPCR, quantitative polymerase chain reaction; SDHA, succinate dehydrogenase complex, subunit A, flavoprotein; TBP, TATA-binding protein.

The mRNA expression for three genes (*TBP*, *HMBS*, *SDHA*) was evaluated to determine the best reference gene. Cq values between EFV- or RMP-treated samples, relative to DMSO-treated samples, were compared for each reference gene (Fig. 1A–C). *TBP* was selected as the best reference gene because expression of all three genes was unaltered by the treatment conditions, but variability in *TBP* expression between the three replicates for both EFV and RMP was minimal as shown by the standard error (Fig. 1A). *TBP* was used for normalization in the subsequent analysis to evaluate the effects of EFV or RMP on mRNA expression of DMEs and NRs.

**FIG. 2.**
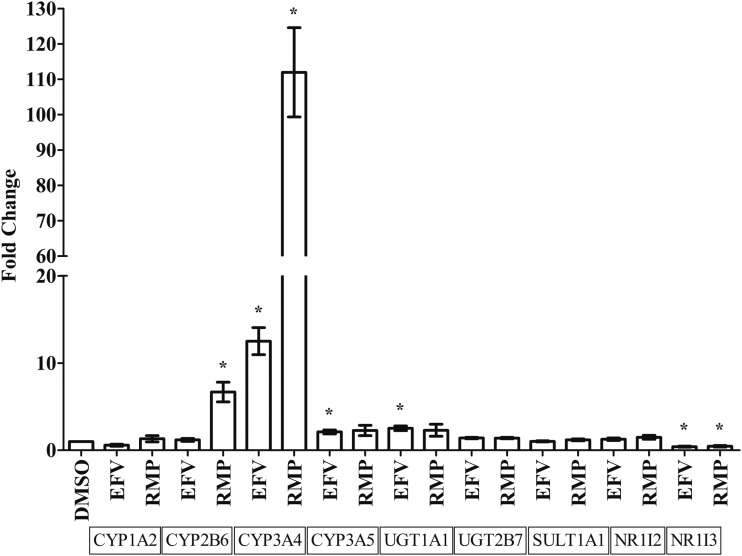
Effects of efavirenz and rifampicin on mRNA expression of genes coding for drug-metabolizing enzymes or nuclear receptors in HepaRG cells (average of three biological replicates) relative to *TBP* as reference gene. Differentiated HepaRG cells were treated with efavirenz (6.4 μM), rifampicin (24.4 μM), or DMSO (0.02%) for 24 h. Expression of mRNA for genes coding for drug-metabolizing enzymes or nuclear receptors, in triplicate, were assessed by using qPCR and the fold change in mRNA expression is compared for each treatment condition. Significant (*p* < 0.05) changes in mRNA expression are indicated with *.

Fold changes in mRNA expression were established for *CYP1A2*, *CYP2B6*, *CYP3A4*, *CYP3A5*, *UGT1A1*, *UGT2B7*, *SULT1A1*, *NR1I2*, and *NR1I3* after treatment with EFV and RMP among three biological replicates. Treatment of HepaRG cells with EFV resulted in a significant mRNA expression increase for *CYP3A4* (12.51-fold, *p* = 0.0018), *CYP3A5* (2.10-fold, *p* = 0.0187), and *UGT1A1* (2.52-fold, *p* = 0.0047), whereas *NR1I3* mRNA expression decreased (0.41-fold, *p* = 0.0196) relative to the DMSO-treated control (0.02%) (Fig. 2). Treatment of HepaRG cells with RMP resulted in a significant increase in *CYP2B6* (6.68-fold, *p* = 0.0074) and *CYP3A4* (111.96-fold, *p* = 0.0009) mRNA expression, whereas *NR1I3* mRNA expression decreased (0.46-fold, *p* = 0.0332) relative to the DMSO-treated control (0.02%) (Fig. 2).

### Normalization of microRNA expression

Two-hundred and forty-one microRNAs were included in quantile normalization and differential expression analysis. Supplementary Figure S2 shows the distribution of C_T_ values for microRNA expression for each sample before and after normalization using the quantile normalization method. MicroRNA expression of replicate samples for EFV, RMP, and DMSO were highly correlated before and after normalization using the quantile normalization method (Table 2).

**Table 2.** Pearson Correlation of MicroRNA Expression Between Replicate Samples Before and After Quantile Normalization

*Replicate samples*	*Pearson correlation coefficient before normalization*	*Pearson correlation coefficient after quantile normalization*
EFV replicate A vs. B	0.99	0.99
EFV replicate A vs. C	0.92	0.92
EFV replicate B vs. C	0.92	0.91
RMP replicate A vs. B	0.96	0.96
RMP replicate A vs. C	0.92	0.92
RMP replicate B vs. C	0.97	0.96
DMSO replicate A vs. B	0.98	0.98
DMSO replicate A vs. C	0.96	0.95
DMSO replicate B vs. C	0.95	0.95

DMSO, dimethyl sulfoxide; EFV, efavirenz; RMP, rifampicin.

### MicroRNAs differentially expressed after treatment with EFV

Limma analysis was used, after quantile normalization, to compare microRNA expression (C_T_ values) for replicate samples treated with EFV to replicate samples treated with DMSO (0.02%) alone. Twenty-four microRNAs were differentially expressed with *p* < 0.05. Expression of miR-622, miR-27a, miR-27b, miR-122#, miR-221, miR-383, miR-548d, miR-29b, miR-22#, and miR-93# was upregulated. Expression of the following microRNAs was downregulated: miR-216b, miR-19a, let-7a, miR-25, miR-422a, miR-885-5p, miR-197, miR-193a-3p, miR-210, miR-30b, miR-876-3p, miR-203, miR-181c, and miR-195.

**FIG. 3.**
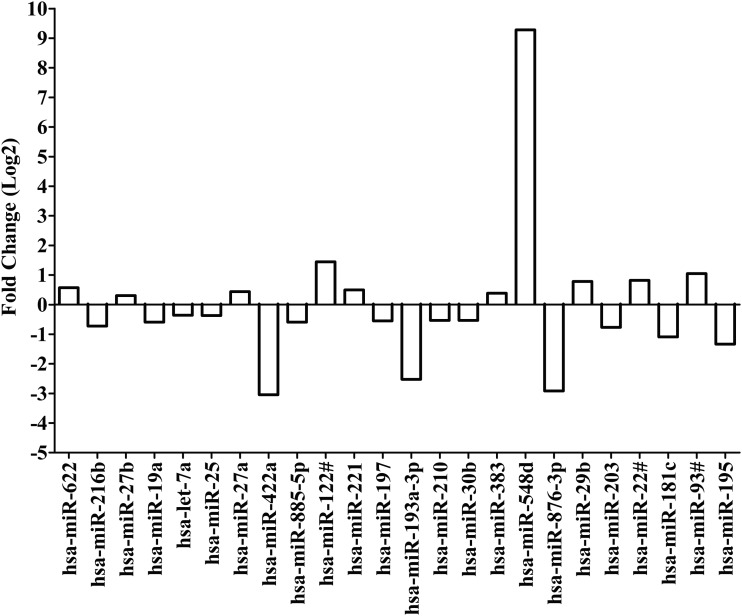
Fold change for differentially expressed microRNAs after treatment with efavirenz based on quantile normalization followed by limma differential expression analysis using the R/Bioconductor package “Automated Analysis of High-Throughput qPCR Data.” Differentiated HepaRG cells were treated with efavirenz (6.4 μM), rifampicin (24.4 μM), or DMSO (0.02%) for 24 h (including three biological replicates). Expression of microRNAs were assessed by using the TaqMan^®^ OpenArray^®^ Human MicroRNA Panel and QuantStudio^™^ 12K Flex system. The fold change in microRNA expression is compared for efavirenz versus DMSO. Quantile normalization is used to minimize variability between TaqMan OpenArray Human MicroRNA Panels and assumes that most microRNAs are not differentially expressed. Limma analysis involves fitting of a one-factorial linear model for each microRNA between efavirenz- and DMSO-treated replicates and the standard errors are moderated using an empirical Bayes model resulting in moderated *t*-statistics for each microRNA.

Interestingly, expression of miR-548d was upregulated by nearly 622-fold in replicate samples treated with EFV. Downregulation of miR-422a, miR-876-3p, and miR-193a-3p were about 0.12-fold, 0.13-fold, and 0.17-fold, respectively. These changes suggest that miR-548d, miR-422a, miR-876-3p, and miR-193a-3p are candidate microRNAs of importance in EFV disposition (Fig. 3 and Supplementary Table S1).

### MicroRNAs differentially expressed after treatment with RMP

**FIG. 4.**
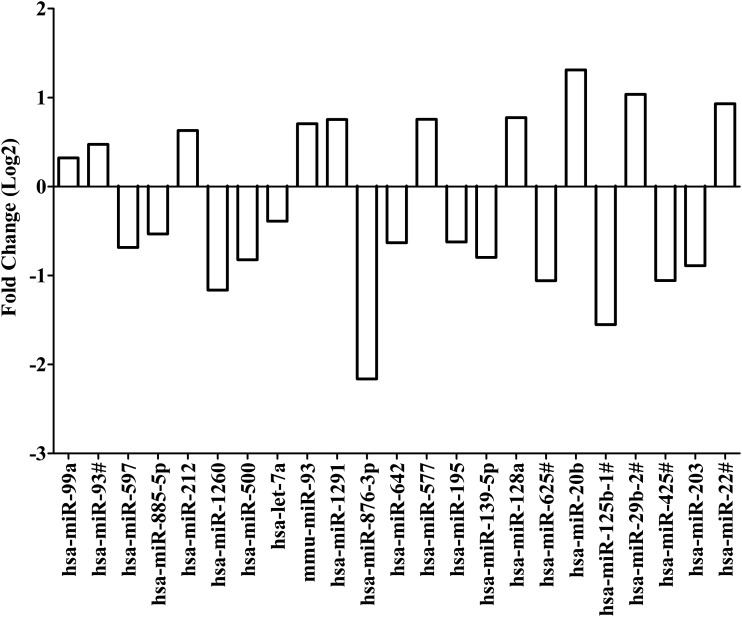
Fold change for differentially expressed microRNAs after treatment with rifampicin based on quantile normalization followed by limma differential expression analysis using the R/Bioconductor package “Automated Analysis of High-Throughput qPCR Data.” Differentiated HepaRG cells were treated with efavirenz (6.4 μM), rifampicin (24.4 μM), or DMSO (0.02%) for 24 h (including three biological replicates). Expression of microRNAs were assessed by using the TaqMan OpenArray Human MicroRNA Panel and QuantStudio 12K Flex system. The fold change in microRNA expression is compared for rifampicin versus DMSO. Quantile normalization is used to minimize variability between TaqMan OpenArray Human MicroRNA Panels and assumes that most microRNAs are not differentially expressed. Limma analysis involves fitting of a one-factorial linear model for each microRNA between rifampicin- and DMSO-treated replicates and the standard errors are moderated using an empirical Bayes model resulting in moderated *t*-statistics for each microRNA.

MicroRNA expression (C_T_ values) was compared for replicate samples treated with RMP and replicate samples treated with DMSO alone. Quantile normalization followed by limma analysis showed 23 differentially expressed microRNAs with *p* < 0.05. Expression of miR-99a, miR-93#, miR-212, mmu-miR-93, miR-1291, miR-577, miR-128a, miR-20b, miR-29b-2#, and miR-22# was upregulated. Expression was downregulated for miR-597, miR-885-5p, miR-1260, miR-500, let-7a, miR-876-3p, miR-642, miR-195, miR-139-5p, miR-625#, miR-125b-1#, miR-425#, and miR-203. Interestingly, downregulation of miR-876-3p and miR-125b-1# was 0.22-fold and 0.34-fold, respectively, in replicate samples treated with RMP. Upregulation of miR-20b was 2.48-fold. MicroRNAs, miR-876-3p, miR-125b-1#, and miR-20b, were identified as candidate microRNAs of importance in RMP disposition (Fig. 4 and Supplementary Table S2).

### Potential target genes of differentially expressed microRNAs

MicroRNAs with differential expression after treatment with EFV or RMP were searched using MiRTarBase and IPA to identify their potential target genes (Tables 3 and 4). For the microRNAs differentially expressed after treatment with EFV: (1) using MiRTarBase, miR-122-5p, and miR-27b-3p both had 10 targets and (2) the microRNA with the most targets predicted by IPA was let-7a-5p (20 targets). For the microRNAs differentially expressed after treatment with RMP: (1) using MiRTarBase, miR-128-3p had 14 targets followed by miR-93-5p with 10 targets and (2) the 2 microRNAs with the most targets predicted by IPA were miR-1291 (26 targets) and let-7a-5p (20 targets). The specific target genes for microRNAs differentially expressed after treatment with EFV or RMP are listed in Supplementary Tables S3 and S4.

**Table 3.** Number of Potential Target Genes for MicroRNAs Differentially Expressed After Exposure to Efavirenz

*MicroRNA*	*MiRTarBase*	*IPA*
hsa-let-7a-5p	9	20
hsa-miR-122-3p	1	4
hsa-miR-122-5p	10	8
hsa-miR-181c-5p	1	7
hsa-miR-193a-3p	1	16
hsa-miR-195-5p	9	17
hsa-miR-197-3p	9	13
hsa-miR-19a-3p	3	8
hsa-miR-203a-3p	4	
hsa-miR-210-3p	2	5
hsa-miR-216b-5p	2	10
hsa-miR-221-3p	2	8
hsa-miR-22-5p	1	8
hsa-miR-25-3p	2	2
hsa-miR-27a-3p	7	11
hsa-miR-27b-3p	10	
hsa-miR-29a-3p	4	
hsa-miR-29b-3p	5	13
hsa-miR-30b-5p	2	9
hsa-miR-383-5p	2	14
hsa-miR-422a	3	15
hsa-miR-548d-3p	3	2
hsa-miR-622	1	9
hsa-miR-876-3p		10
hsa-miR-885-5p	2	4
hsa-miR-93-3p	1	6

IPA, Ingenuity Pathway Analysis.

**Table 4.** Number of Potential Target Genes for MicroRNAs Differentially Expressed After Exposure to Rifampicin

*MicroRNA*	*MiRTarBase*	*IPA*
hsa-let-7a-5p	9	20
hsa-miR-125b-1-3p	1	4
hsa-miR-1260a	4	14
hsa-miR-128-3p	14	15
hsa-miR-1291	1	26
hsa-miR-139-5p		3
hsa-miR-195-5p	9	17
hsa-miR-203a-3p	4	
hsa-miR-20b-5p	7	11
hsa-miR-212-3p	2	8
hsa-miR-22-5p	1	8
hsa-miR-29b-2-5p	1	7
hsa-miR-425-3p		2
hsa-miR-500a	5	3
hsa-miR-577	3	3
hsa-miR-597-5p		7
hsa-miR-625-3p		2
hsa-miR-642a-5p	1	4
hsa-miR-876-3p		10
hsa-miR-885-5p	2	4
hsa-miR-93-3p	1	6
hsa-miR-93-5p	10	
hsa-miR-99a-3p	1	5
hsa-miR-99a-5p		1

## Discussion

In this study, the effects of EFV or RMP treatment *in vitro* on mRNA and microRNA expression were assessed using HepaRG cells. Differentiated HepaRG cells were used as an *in vitro* model alternative to primary human hepatocytes because mRNA expression and induction of DMEs are comparable to that of hepatocytes, yet HepaRG cells have an extended lifespan (Andersson, 2010; Aninat et al., 2006). To ensure clinically relevant (yet not toxic) EFV and RMP concentrations and minimize the amount of DMSO used, the concentrations were selected as 6.4 μM EFV, 24.4 μM RMP, and 0.02% DMSO as solvent. In an earlier study using four cell lines, 10 to 20 μM EFV reduced the rate of proliferation, after 96 h of treatment, by ∼50% and fully rescinded proliferation at 40–50 μM EFV (Sciamanna et al., 2013). Treatment of HepaRG cells with DMSO for 2 weeks have been reported to reduce total cellular protein content and increase cell leakage (Hoekstra et al., 2011).

RMP is a typical inducer of DMEs and often used as positive control in *in vitro* studies. RMP is known to be a potent activator of the NR, PXR. PXR functions as a sensor of endobiotic and xenobiotic substances and regulates transcription of many target genes, including *CYP1A2, CYP2B6, CYP3A4,* and *UGT1A1* (Chai et al., 2013) in response to xenobiotic substances. The increases in *CYP2B6, CYP3A4, CYP3A5,* and *UGT1A1* mRNA expression, although not significant for *CYP3A5* and *UGT1A1*, observed in this study after treatment with RMP agree with previous studies using HepaRG cells and hepatocytes (Antherieu et al., 2010; Burk et al., 2004; Gerets et al., 2012; Higuchi et al., 2016; Templeton et al., 2011). After treatment with EFV, mRNA expression of *CYP3A4*, *CYP3A5*, and *UGT1A1* increased and agrees with what have been reported in studies using hepatocytes and other cell-based *in vitro* induction models (Blas-Garcia et al., 2010; Hariparsad et al., 2008; Kamiguchi et al., 2010; Mugundu et al., 2010).

Induction of mRNA expression of the abovementioned enzymes is controlled by multiple members of the NR family. Similarly, to PXR, the constitutive androstane receptor (CAR) is also a NR that functions as a sensor of endobiotic and xenobiotic substances. The target genes of PXR overlap with those of CAR because both PXR and CAR interact with the same response elements in target gene promoters (Chen et al., 2005; Smirlis et al., 2001). CAR activation, in response to endobiotic and xenobiotic substances, alters mRNA expression of *CYP2A6*, *CYP2B6*, *CYP2C9*, *CYP2C19*, *CYP3A4*, *UGT1A1*, and *ABCB1* (Burk et al., 2005; Gerbal-Chaloin et al., 2002).

EFV has been reported to preferentially induce *CYP2B6* mRNA expression through direct interaction and activation of CAR (Faucette et al., 2007; Meyer zu Schwabedissen et al., 2012). In this study, *CYP2B6* mRNA expression was not increased as expected after treatment with EFV, but expression of *NR1I3* (CAR) was decreased.

Furthermore, *CYP1A2* mRNA expression was decreased following treatment with EFV, although not significantly. Induction of *CYP1A2* mRNA expression is influenced by multiple NRs and transcription factors, including AHR, PXR, CAR, HNF1α, HNF4α, and PPARG (Maglich et al., 2002; Martinez-Jimenez et al., 2006; Narvaez et al., 2005; Nebert et al., 2004; Okey et al., 2005; Yoshinari et al., 2010). The decrease in *CYP1A2* mRNA expression might be a consequence of the decrease in *NR1I3* (CAR) expression, although expression of other transcription factors was not evaluated. Several possible reasons exist for the observed decrease in *NR1I3* mRNA expression.

A glucocorticoid response element is present in the distal region of *NR1I3* (CAR) promoter in hepatocytes, suggesting transcriptional activation of *NR1I3* by glucocorticoid receptor and is altered in the presence of dexamethasone and glucocorticoids (Pascussi et al., 2003). Alternatively, an increased level of the proinflammatory cytokine IL-6 has been reported to decrease *NR1I3* mRNA expression (Pascussi et al., 2000). MicroRNA regulation can also affect *NR1I3* mRNA expression. A negative feedback loop exists between miR-137 and *NR1I3*, where miR-137 downregulates *NR1I3* mRNA expression through targeting in the 3′-UTR and CAR downregulates miR-137 expression (Takwi et al., 2014). These molecular mechanisms suggest that *NR1I3* mRNA expression is tightly controlled and likely a limiting factor of DME induction.

We identified that 10 microRNAs were upregulated, and 13 microRNAs were downregulated after treatment with 24.4 μM RMP. Several previous studies investigated the effects of RMP on microRNA expression in hepatocytes and HepaRG cells (Benson et al., 2016; Li et al., 2015, 2016; Ramamoorthy et al., 2013; Smith et al., 2014; Yan et al., 2017). In the study by Yan et al. (2017), HepaRG cells were treated with 10 μM RMP and the expression of 18 microRNAs was increased, whereas the expression of 72 microRNAs was decreased. Like our findings, after treatment with RMP, the expression of miR-29b-2-5p was upregulated. The other differentially expressed microRNAs identified by Yan et al. (2017), but not in our study, are likely because of using different concentrations of RMP and DMSO.

None of the microRNAs, found to be differentially expressed after treatment with RMP in this study, was reported as differentially expressed by Benson et al. (2016) (reanalyses of the data by Ramamoorthy et al., 2013) using human hepatocytes. Likely reasons for differences in our findings as compared with that of Ramamoorthy et al. (2013) and Benson et al. (2016) include differences in microRNA expression between hepatocytes and HepaRG cells, the use of 10 μM RMP, the use of methanol as solvent, and interdonor variability in microRNA expression in human hepatocytes as seven donors were treated as biological replicates.

MicroRNA-22, miR-20b, and miR-212 were upregulated in our study, whereas miR-22 and miR-20b were upregulated but miR-212 was downregulated in the study by Takahashi et al. (2014). In the study by Takahashi et al. (2014), human hepatocytes from 10 donors were treated with 10 μM RMP (dissolved in DMSO) for 48 h and despite using the same concentration and time of treatment with RMP as in the study by Benson et al. (2016), none of the differentially expressed microRNAs in human hepatocytes overlaps.

This is the first study to identify microRNAs differentially expressed after treatment with EFV in a hepatic *in vitro* cell model. Ten microRNAs were upregulated, whereas 14 microRNAs were downregulated after treatment with EFV. MicroRNA-181c and miR-25 were downregulated following treatment with EFV in this study and in the study by Sciamanna et al. (2013). Cell type-specific microRNA expression is a probable reason for the differences in differentially expressed microRNAs in this study using HepaRG cells compared with A-375 melanoma cells (Sciamanna et al., 2013).

The study by Jin et al. (2016) used human hepatocytes and HepaRG cells to demonstrate an inverse correlation between miR-25-3p and *CYP2B6* expression, binding of miR-25-3p to the 3′-UTR of *CYP2B6*, suppression of *CYP2B6* expression through overexpression of miR-25-3p, and decreased RMP-dependent induction of *CYP2B6* mRNA and protein. The observed decrease in expression of miR-25 after treatment with EFV in this study suggests microRNA regulation of *CYP2B6* induction after treatment with EFV in a manner like RMP. *CYP2C19* and *NAT1* are listed in MiRTarBase as targets of miR-25-3p and decreased expression of miR-25-3p likely also affects expression of *CYP2C19* and *NAT1*. Both miR-27a and miR-27b are upregulated following treatment of HepaRG cells with EFV and both microRNAs are known to modify mRNA expression of *CYP3A4* (Pan et al., 2009; Shi et al., 2015).

The observed upregulation of these two microRNAs point toward a potential control mechanism to lower *CYP3A4* mRNA expression after induction by EFV. Additional target genes of miR-27a and miR-27b listed in MiRTarBase include *DPYD*, *ABCA1*, *ALDH9A1*, *CYP1B1*, *PPARG*, *ATP7B*, and *SLC5A6*. Expression of *ARNT*, *CYP1B1*, *AHR*, *ALDH1B1*, *ALDH5A1*, *SLC16A1*, *SLC29A2*, *SLC29A1*, *SLC2A4*, and *SLCO3A1* could (as in MiRTarBase) be decreased because of the upregulation of miR-122, miR-221-3p, miR-29b-3p, miR-383-5p, miR-548d-3p, and miR-622. Our study shows that treatment of HepaRG cells with EFV alters the expression of multiple microRNAs and prioritized multiple DMEs and transporters, whose expression could be altered through microRNA regulation.

Overlap exists in the microRNAs that are differentially expressed after treatment with EFV or RMP. Expression of miR-22 and miR-93 is upregulated, while expression of miR-203, miR-195, miR-876-3p, let-7a, and miR-885-5p is downregulated after treatment with both medicines. Based on prediction by IPA, expression of *CYP4Z1*, *GSTM2*, *GSTT2/GSTT2B*, and *GPX1* could be altered because of the increased expression of miR-22 and miR-93. As listed in MiRTarBase, expression of *PDE3A* and *CBR1* would be altered in response to upregulation of both microRNAs.

Downregulation of miR-203, miR-195, miR-876-3p, let-7a and miR-885-5p would affect expression of 20 genes (according to MiRTarBase) and 51 genes (according to IPA) involved in pharmacokinetics and pharmacodynamics. Identification of microRNAs of which expression is altered after treatment with multiple medications could identify the suite of microRNAs that regulate the genes coding for enzymes, transporters, and NRs involved in disposition of medicines.

Many previous pharmacogenomic studies in individuals with HIV/AIDS receiving EFV-containing treatment have reported that some patients have EFV plasma concentrations unexplained by the CYP2B6*6 and *18 variants. Genetic variants in *CYP2B6* other than *6 and *18 and genetic variants in other genes involved in EFV disposition may be a reason for the unexpectedly high or low EFV plasma concentrations. Inducers of *CYP2B6* gene expression may result in lower-than-expected EFV plasma concentrations. Additionally, microRNAs with altered expression in response to EFV may contribute to why certain individuals have high EFV plasma concentrations but do not have the CYP2B6*6 or *18 variants, or why certain individuals have low EFV plasma concentrations but are carriers of the CYP2B6 *6 or *18 variants.

MicroRNAs that are upregulated following treatment with EFV, including miR-93#, miR-22#, miR-29b, miR-548d, miR-383, miR-221, miR-122#, miR-27a (experimentally confirmed to target *CYP3A4*; Shi et al., 2015), miR-27b (experimentally confirmed to target *CYP3A4*; Pan et al., 2009), and miR-622, may result in suppression of expression of a target gene that metabolizes EFV and potentially cause higher-than-expected EFV plasma concentrations. MicroRNAs that are downregulated following treatment with EFV, including miR-195, miR-181c, miR-203, miR-876-3p, miR-30b, miR-210, miR-193a-3p, miR-197, miR-885-5p, miR-422a, miR-25 (experimentally confirmed to target *CYP2B6* (Jin et al., 2016)), let-7a, miR-19a, and miR-216b may result in loss of suppression of expression of a target gene and, subsequently, lower-than-expected EFV plasma concentrations.

However, for a microRNA to contribute to interindividual variability in EFV plasma concentrations, the microRNA needs to act differently between individuals. This could occur if a microRNA is upregulated after treatment with EFV in some individuals but not others, or if a microRNA is downregulated after treatment with EFV in some individuals, or if interaction of a microRNA with a target gene is abolished in some individuals, or if interaction of a microRNA with a target gene is created in some individuals. Further studies are necessary to completely understand how microRNA regulation differ between individuals and how these differences influence response to medicines.

### Clinical implications

This study assessed the effects of EFV and RMP on microRNA and mRNA expression in a hepatic *in vitro* cell model and has clinical implications. In Africa, patients are often treated for HIV/AIDS and tuberculosis simultaneously and because the same enzymes are responsible for metabolism of EFV and RMP drug/drug interactions may occur. In this study, treatment with EFV and RMP increased mRNA expression of *CYP3A4*, *CYP3A5*, and *UGT1A1*, yet decreased mRNA expression of *NR1I3,* which codes for the CAR. *NR1I3* is a key regulator of many DMEs and transporters and decreased expression of CAR can, thus, have a large impact on xenobiotic and endobiotic metabolism. RMP is a typical inducer of DMEs and the increase in expression of DMEs could potentially result in increased metabolism of EFV and subsequently, subtherapeutic EFV plasma concentrations, treatment failure, and switching to more expensive antiretroviral medicines.

Similarly, it is important to optimize treatment with RMP shortly after starting with therapy because treating patients with drug-resistant tuberculosis is costly. It is, thus, crucial to have optimal therapeutics and limit treatment failure in a resource-limited setting burdened by the HIV/AIDS and tuberculosis epidemics, like Africa.

Furthermore, the expression of several microRNAs (e.g., expression of miR-22 and miR-93 was upregulated after treatment with both medicines; expression of miR-203, miR-195, miR-876-3p, let-7a, and miR-885-5p were downregulated after treatment with both medicines) was altered by treatment with both EFV and RMP. These microRNAs likely regulate mRNA expression of the same genes and may impact the rate of metabolism for both EFV and RMP.

However, it is not known what the impact of altered microRNA expression, after treatment with EFV and RMP, is on pharmacokinetic and pharmacodynamic outcomes in patients with either HIV/AIDS or tuberculosis and coinfected patients. Pharmacogenomic studies that evaluate the predictability of pharmacokinetic and pharmacodynamic outcomes, in patients, based on genetic variants need to also consider the impact of altered microRNA expression. This study identified candidate microRNAs with altered expression, following treatment with EFV and RMP, to consider in future pharmacogenomic studies.

## Conclusions

Differentiated HepaRG cells were used to show changes in expression of several genes and microRNAs following treatment with EFV and RMP. Although previous studies have identified candidate microRNAs with altered expression in response to RMP in hepatocytes or HepaRG cells, the changes in microRNA expression after treatment with EFV have not been studied in a hepatic *in vitro* cell model. The microRNAs identified to be up- or downregulated, after treatment with RMP or EFV, are predicted to target genes involved in disposition of EFV or RMP and other medicines and could influence the level of expression of these genes and, subsequently, how an individual respond to medicines.

For example, miR-25-3p has been shown to suppress *CYP2B6* expression (Jin et al., 2016) and is downregulated after treatment with EFV (this study), which could affect EFV metabolism by CYP2B6. Experimental validation of the potential microRNA target genes is necessary to determine if the differentially expressed microRNAs have a direct impact on mRNA expression following treatment with EFV or RMP. Pharmacogenomic studies have focused largely on how genetic variant in genes involved in disposition of medicines affects response to medicines. Our study shows that differential expression of microRNAs may contribute to the complexity of disposition of RMP and EFV. Future studies are needed to incorporate the impact of microRNAs in pharmacogenomic algorithms to narrow variability in response to medicines.

## Supplementary Material

Supplemental data

Supplemental data

Supplemental data

Supplemental data

Supplemental data

## Abbreviations Used

CAR: constitutive androstane receptor
cDNA: complementary DNA
DMEs: drug-metabolizing enzymes
DMSO: dimethyl sulfoxide
EFV: efavirenz
HMBS: hydroxymethylbilane synthase
IPA: Ingenuity Pathway Analysis
mRNA: messenger RNA
NRs: nuclear receptors
PXR: pregnane X receptor
qPCR: quantitative polymerase chain reaction
RMP: rifampicin
SDHA: succinate dehydrogenase complex, subunit A, flavoprotein
TBP: TATA-binding protein
UTR: untranslated region
