# Supplementary material for: MicroRNA Mediated Changes in Drug Metabolism and Target Gene Expression by Efavirenz and Rifampicin In Vitro: Clinical Implications
Source: OMICS. 2019 Oct 4;23(10):496–507. doi: 10.1089/omi.2019.0122 (PMC6806364; doi:10.1089/omi.2019.0122)
Supplement: Supplemental data [file Supp_FigureS1.pdf]

Supplementary Data

A

B

C

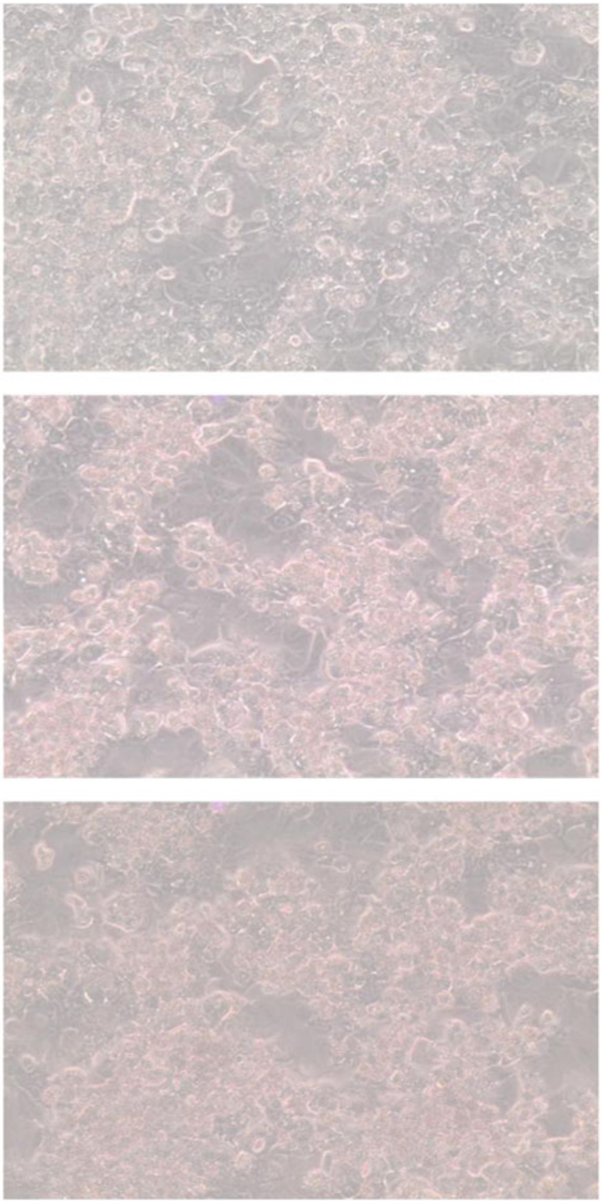

SUPPLEMENTARY FIG. S1. Representative images showing no effects of DMSO and drug treatments (efavirenz and rifampicin) on HepaRG cell morphology after 24 h at 20× magnification. **(A)** HepaRG cells treated with 0.02% DMSO; **(B)** HepaRG cells exposed to efavirenz (6.4 μM); **(C)** HepaRG cells exposed to rifampicin (24.4 μM). DMSO, dimethyl sulfoxide.
