# Supplementary material for: MicroRNA Mediated Changes in Drug Metabolism and Target Gene Expression by Efavirenz and Rifampicin In Vitro: Clinical Implications
Source: OMICS. 2019 Oct 4;23(10):496–507. doi: 10.1089/omi.2019.0122 (PMC6806364; doi:10.1089/omi.2019.0122)
Supplement: Supplemental data [file Supp_FigureS2.pdf]

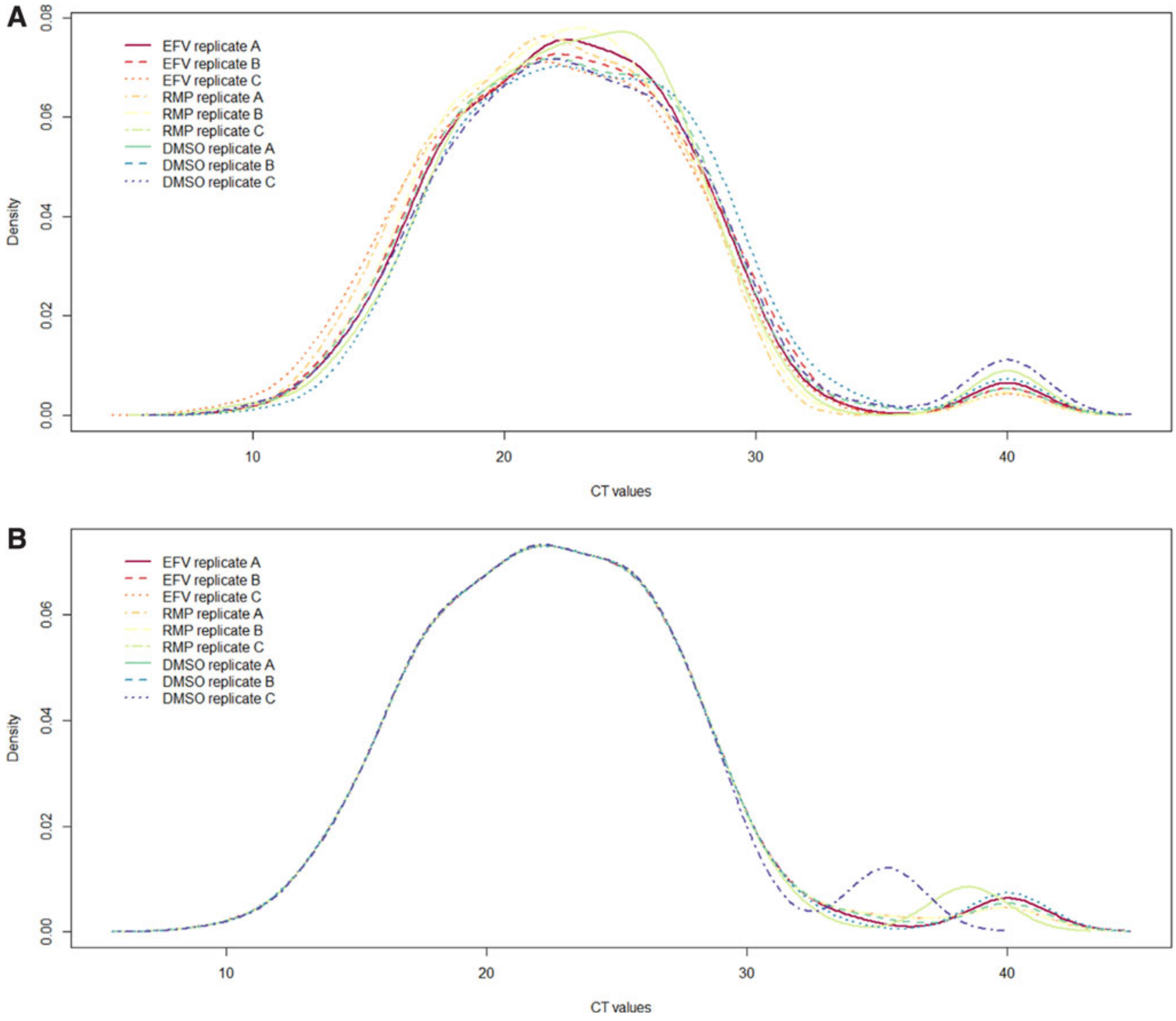

SUPPLEMENTARY FIG. S2. Changes in distribution of C_T values of microRNA expression before and after quantile normalization. **(A)** Distribution of C_T values before normalization; **(B)** distribution of C_T values after quantile normalization.
