# Supplementary material for: MicroRNA Mediated Changes in Drug Metabolism and Target Gene Expression by Efavirenz and Rifampicin In Vitro: Clinical Implications
Source: OMICS. 2019 Oct 4;23(10):496–507. doi: 10.1089/omi.2019.0122 (PMC6806364; doi:10.1089/omi.2019.0122)
Supplement: Supplemental data [file Supp_TableS1-S2.pdf]

Supplementary Table S1. Differentially Expressed MicroRNAs After Treatment with Efavirenz Using Quantile Normalization and limma Analysis

Differentially expressed microRNAs	*Unadjusted* p-*value*	*Benjamini/Holm adjusted* p-*value*	*Fold change*
hsa-miR-195	0.000337276459063447	0.0515786962536653	0.396432775290664
hsa-miR-93#	0.00094252215604033‌5	0.0515786962536653	2.06564536859351
hsa-miR-181c	0.000982899486009876	0.0515786962536653	0.469472029289138
hsa-miR-22#	0.00107009743264866	0.0515786962536653	1.76255303254325
hsa-miR-203	0.00382764191965445	0.15374361710612‌1	0.587939960004118
hsa-miR-29b	0.00476496657292314	0.16405099201064	1.72278269924197
hsa-miR-876-3p	0.00718623839695659	0.216485431708317	0.132256213447353
hsa-miR-548d	0.0098394947687518	0.263479804363243	621.891254366142
hsa-miR-383	0.0130866556255003	0.315388400574557	1.3077720644938
hsa-miR-30b	0.0157387017838264	0.343467471893807	0.690424495398716
hsa-miR-210	0.0187419217968033	0.347446396386892	0.689804410956658
hsa-miR-193a-3p	0.0209508305696285	0.359145184133895	0.173869173414376
hsa-miR-197	0.0226467075579982	0.359145184133895	0.683827195189982
hsa-miR-221	0.0238436636769391	0.359145184133895	1.41055142060532
hsa-miR-122#	0.0257091138252294	0.361853384863971	2.72514563888616
hsa-miR-885-5p	0.0270263938902551	0.361853384863971	0.662911823131997
hsa-miR-422a	0.0332292593349066	0.406836787743004	0.121127016829242
hsa-miR-27a	0.0337623890243157	0.406836787743004	1.35260513953923
hsa-miR-25	0.0428679221017306	0.443614181560503	0.773742768520296
hsa-let-7a	0.0429671317990044	0.443614181560503	0.780105278894354
hsa-miR-19a	0.0447457123801481	0.443614181560503	0.664496420586468
hsa-miR-27b	0.0450616434097777	0.443614181560503	1.236624426065
hsa-miR-216b	0.0460180686266081	0.443614181560503	0.604329556215936
hsa-miR-622	0.0484155942748276	0.448775316162825	1.48631586229885

Supplementary Table S2. Differentially Expressed MicroRNAs After Treatment with Rifampicin Using Quantile Normalization and limma Analysis

Differentially expressed microRNAs	*Unadjusted* p-*value*	*Benjamini/Holm adjusted* p-*value*	*Fold change*
hsa-miR-22#	0.000492154762165706	0.118609297681935	1.90654814481948
hsa-miR-203	0.00167085881913639	0.201338487705935	0.539309437481509
hsa-miR-425#	0.00666464240690603	0.409208432368125	0.480642803154659
hsa-miR-29b-2#	0.0110343263364336	0.409208432368125	2.05185093725171
hsa-miR-125b-1#	0.0113656209862097	0.409208432368125	0.341229626224244
hsa-miR-20b	0.0121437333199812	0.409208432368125	2.480816135972
hsa-miR-625#	0.0124533427596102	0.409208432368125	0.48029743197674
hsa-miR-128a	0.0135836824022614	0.409208432368125	1.71119005136521
hsa-miR-139-5p	0.0196115340325911	0.427138625325539	0.575884681420726
hsa-miR-195	0.0199352066620266	0.427138625325539	0.649235959705979
hsa-miR-577	0.0231811088640087	0.427138625325539	1.68797822847066
hsa-miR-642	0.0234346951794707	0.427138625325539	0.645546351447115
hsa-miR-876-3p	0.0277549132096698	0.427138625325539	0.223440554323334
hsa-miR-1291	0.0289507256434038	0.427138625325539	1.6856830895094
mmu-miR-93	0.0307026844036966	0.427138625325539	1.63119123226095
hsa-let-7a	0.031364872002381	0.427138625325539	0.763462726769337
hsa-miR-500	0.0319024699413266	0.427138625325539	0.564960622997247
hsa-miR-1260	0.03802433990887	0.433210303965054	0.446307857416551
hsa-miR-212	0.0390896042842577	0.433210303965054	1.54744581031954
hsa-miR-885-5p	0.0407928620218925	0.433210303965054	0.69102739688754
hsa-miR-597	0.0413437219551711	0.433210303965054	0.622117614141279
hsa-miR-93#	0.0440721946576306	0.442558288020374	1.38852731262891
hsa-miR-99a	0.0463233767522586	0.446557351891773	1.249982075793
