# Supplementary material for: MicroRNA Mediated Changes in Drug Metabolism and Target Gene Expression by Efavirenz and Rifampicin In Vitro: Clinical Implications
Source: OMICS. 2019 Oct 4;23(10):496–507. doi: 10.1089/omi.2019.0122 (PMC6806364; doi:10.1089/omi.2019.0122)
Supplement: Supplemental data [file Supp_TableS3.pdf]

Supplementary Table S3. Target Genes According to the MiRTarBase Database

MicroRNA	*MiRTarBase target*
Differentially expressed microRNAs after treatment with EFV	
hsa-let-7a-5p	AHR; ADH5; ALDH7A1; FMO4; NR1I2; SLC5A6; CYP2R1; SOD2
hsa-miR-122-3p	ALDH5A1
hsa-miR-122-5p	CYP3A5; SLC15A2; CYP7A1; GSTM3; SLC7A5; CYP20A1; CHST12; CHST3; ATP7A
hsa-miR-181c-5p	ALDH9A1
hsa-miR-193a-3p	SLC7A5
hsa-miR-195-5p	SLC7A5; UGT2B4; ABCB7; ABCC6; ALDH3B1; SLC29A1; SLCO3A1
hsa-miR-197-3p	ABCC3; CES1; GSTO1; GSTK1; GSTM5; HNF4A; SOD1; SOD2
hsa-miR-19a-3p	ABCA1; SLC27A1; PPARA
hsa-miR-203a-3p	GSTO2; CYP20A1; SOD2
hsa-miR-210-3p	ALDH5A1; SLCO3A1
hsa-miR-216b-5p	ABCC12; UGT2B28
hsa-miR-221-3p	ARNT; CYP1B1
hsa-miR-22-5p	PDE3A
hsa-miR-25-3p	CYP2C19; NAT1
hsa-miR-27a-3p	DPYD; ABCA1; ALDH9A1; CYP1B1; PPARG; ATP7B
hsa-miR-27b-3p	CYP3A4; DPYD; ABCA1; CYP1B1; PPARG; SLC5A6; ATP7B
hsa-miR-29b-3p	EPHX2; PPARD; SLC16A1; SLC29A2
hsa-miR-30b-5p	SLC7A5; CAT
hsa-miR-383-5p	AHR; ALDH1B1
hsa-miR-422a	CYP7A1; CYP20A1; CYP8B1
hsa-miR-548d-3p	SLC29A1; SLC2A4
hsa-miR-622	SLCO3A1
hsa-miR-885-5p	SULT1B1; SOD2
hsa-miR-93-3p	CBR1
Differentially expressed microRNAs after exposure to RMP	
hsa-let-7a-5p	AHR; ADH5; ALDH7A1; FMO4; NR1I2; SLC5A6; CYP2R1; SOD2
hsa-miR-125b-1-3p	SOD2
hsa-miR-1260a	SLC7A5; SLC27A1; GSTCD; MGST3
hsa-miR-128-3p	ALDH4A1; ABCA1; ABCG1; ALDH9A1; CYP20A1; RXRA; SLC16A1; SLC22A14; PDE3A; PDE3B
hsa-miR-1291	ABCC1
hsa-miR-195-5p	SLC7A5; UGT2B4; ABCB7; ABCC6; ALDH3B1; SLC29A1; SLCO3A1
hsa-miR-203a-3p	GSTO2; CYP20A1; SOD2
hsa-miR-20b-5p	ABCA1; ALDH9A1; PPARG; SLC28A1; SLCO5A1; SOD2
hsa-miR-212-3p	CYP20A1; SOD2
hsa-miR-22-5p	PDE3A
hsa-miR-29b-2-5p	SLC7A5
hsa-miR-500a-3p	UGT2B10; SOD2
hsa-miR-500a-5p	GSTCD
hsa-miR-577	SLC5A6; SOD2
hsa-miR-642a-5p	SLC22A6
hsa-miR-885-5p	SULT1B1; SOD2
hsa-miR-93-3p	CBR1
hsa-miR-93-5p	ABCA1; ALDH9A1; SLC19A1; SLC28A1; SLC29A2; SLC2A4; SLCO5A1; SOD2; PDE3B
hsa-miR-99a-3p	GSTM5

EFV, efavirenz; RMP, rifampicin.
