# Supplementary material for: MicroRNA Mediated Changes in Drug Metabolism and Target Gene Expression by Efavirenz and Rifampicin In Vitro: Clinical Implications
Source: OMICS. 2019 Oct 4;23(10):496–507. doi: 10.1089/omi.2019.0122 (PMC6806364; doi:10.1089/omi.2019.0122)
Supplement: Supplemental data [file Supp_TableS4.pdf]

Supplementary Table S4. Potential Target Genes Predicted by Ingenuity Pathway Analysis

MicroRNA	*IPA target (level of confidence for prediction)*
Differentially expressed microRNAs after treatment with EFV	
hsa-let-7a-5p	*UGT8* (experimentally observed); *CYP19A1* (high); *CYP4F2* (high); *FMO2* (high); *FMO4* (high); *KCNJ11* (high); *ABCC10* (moderate); *ABCC5* (moderate); *CHST3* (moderate); *CHST5* (moderate); *CYP46A1* (moderate); *GPX3* (moderate); *GPX7* (moderate); *NR1I2* (moderate); *SLC10A1* (moderate); *SLC10A2* (moderate); *SLC13A3* (moderate); *SLC5A6* (moderate); *SLCO2A1* (moderate); *SLCO5A1* (moderate)
hsa-miR-122-3p	*ABCC5* (moderate); *ALDH9A1* (moderate); *SLC22A15* (moderate); *SLC28A2* (moderate)
hsa-miR-122-5p	*GPX7* (experimentally observed); *CDA* (high); *CHST12* (high); *CYP7B1* (moderate); *GPX3* (moderate); *SLC15A1* (moderate); *SLC7A5* (moderate); *SLCO5A1* (moderate)
hsa-miR-181c-5p	*METAP1* (high); *CYP2R1* (moderate); *CYP3A5* (moderate); *FMO5* (moderate); *HSD11B1* (moderate); *SLC22A2* (moderate); *SULT1C2* (moderate)
hsa-miR-193a-3p	*SLC15A1* (high); *ALDH9A1* (moderate); *CHST3* (moderate); *CHST9* (moderate); *CYP11B2* (moderate); *CYP2S1* (moderate); *GSR* (moderate); *KCNJ11* (moderate); *MPO* (moderate); *NOS1* (moderate); *SLC22A8* (moderate); *SLC7A5* (moderate); *SLCO1A2* (moderate); *SLCO2A1* (moderate); *SULT1B1* (moderate); *SULT4A1* (moderate)
hsa-miR-195-5p	*GSTM4* (experimentally observed); *CBR3* (high); *GSTCD* (high); *ABCB5* (moderate); *ALDH1A3* (moderate); *ATP7A* (moderate); *CYP2S1* (moderate); *CYP7B1* (moderate); *GPX1* (moderate); *GSTT2/GSTT2B* (moderate); *KCNJ11* (moderate); *NOS1* (moderate); *PDE3B* (moderate); *SLC13A1* (moderate); *SLC22A17* (moderate); *SLC28A1* (moderate); *XDH* (moderate)
hsa-miR-197-3p	*CBR1* (high); *CYP3A5* (high); *ABCB11* (moderate); *ALDH9A1* (moderate); *CHST2* (moderate); *CHST6* (moderate); *CYP20A1* (moderate); *DHRS1* (moderate); *EPHX2* (moderate); *GSTM5* (moderate); *IAPP* (moderate); *SULF1* (moderate); *SULT4A1* (moderate)
hsa-miR-19a-3p	*CHST1* (high); *DHRS3* (high); *PON2* (high); *ABCA1* (moderate); *ABCB7* (moderate); *GSTM2* (moderate); *SLC6A6* (moderate); *SULF1* (moderate)
hsa-miR-210-3p	*ALDH5A1* (moderate); *CHST1* (moderate); *PDE3A* (moderate); *SLC6A6* (moderate); *SLCO4C1* (moderate)
hsa-miR-216b-5p	*UGT2B28* (high); *CYP3A5* (moderate); *CYP7A1* (moderate); *SLC13A1* (moderate); *SLC16A1* (moderate); *SLC22A15* (moderate); *UGT2B10* (moderate); *UGT2B11* (moderate); *UGT2B4* (moderate); *UGT2B7* (moderate)
hsa-miR-221-3p	*SOD2* (experimentally observed); *ADHFE1* (high); *HNMT* (high); *ALDH1A1* (moderate); *CYP1B1* (moderate); *CYP7A1* (moderate); *PDE3A* (moderate); *UGT2B15* (moderate)
hsa-miR-22-5p	*CYP4Z1* (high); *GSTM2* (high); *GSTT2/GSTT2B* (high); *ABCC1* (moderate); *CYP1A1* (moderate); *GPX5* (moderate); *SLCO1C1* (moderate); *TPMT* (moderate)
hsa-miR-25-3p	*CHST1* (high); *CHST7* (high)
hsa-miR-27a-3p	*CYP1B1* (experimentally observed); *RXRA* (experimentally observed); *PPARG* (experimentally observed); *ABCA1* (moderate); *ALDH4A1* (moderate); *AOX1* (moderate); *CHST1* (moderate); *CYP39A1* (moderate); *SLC16A1* (moderate); *UGT2B28* (moderate); *UGT8* (moderate)
hsa-miR-29b-3p	*ABCB6* (high); *GPX7* (high); *GSTA4* (high); *SLC22A7* (high); *ADH1B* (moderate); *ATP7A* (moderate); *CYP2B6* (moderate); *CYP4F3* (moderate); *DHRS2* (moderate); *FMO1* (moderate); *GSTO2* (moderate); *SLC16A1* (moderate); *XDH* (moderate)
hsa-miR-30b-5p	*ALDH2* (high); *CYP24A1* (high); *SLCO6A1* (high); *CAT* (moderate); *CHST1* (moderate); *CHST2* (moderate); *PON2* (moderate); *SLC22A5* (moderate); *SLC6A6* (moderate)
hsa-miR-383-5p	*CYP3A5* (high); *DHRS13* (high); *ABCC11* (moderate); *ATP7B* (moderate); *CHST10* (moderate); *CYP27B1* (moderate); *CYP4F11* (moderate); *GPX5* (moderate); *GPX6* (moderate); *GSTK1* (moderate); *SLC22A11* (moderate); *SLC22A5* (moderate); *SLC2A5* (moderate); *SULT2A1* (moderate)
hsa-miR-422a	*CHST2* (high); *ADHFE1* (moderate); *ALDH1B1* (moderate); *CDA* (moderate); *CHST3* (moderate); *CYP11B2* (moderate); *CYP7B1* (moderate); *CYP8B1* (moderate); *DDO* (moderate); *GSTM4* (moderate); *GSTO2* (moderate); *NOS3* (moderate); *SLC22A16* (moderate); *SLC7A7* (moderate); *SULF1* (moderate)
hsa-miR-548d-3p	*DHRS7C* (moderate); *SULT1B1* (moderate)
hsa-miR-622	*ABCC12* (high); *ALDH6A1* (moderate); *CYP11B1* (moderate); *DDO* (moderate); *DHRS7B* (moderate); *GSTM5* (moderate); *PPARD* (moderate); *SLC22A18* (moderate); *SLC22A8* (moderate)
hsa-miR-876-3p	*CYP3A5* (high); *ALDH1B1* (moderate); *ALDH3B1* (moderate); *CHST6* (moderate); *CYP27B1* (moderate); *CYP2B6* (moderate); *CYP2C18* (moderate); *PPARG* (moderate); *SLC22A12* (moderate); *SLC22A13* (moderate)
hsa-miR-885-5p	*TPMT* (high); *ALDH1B1* (moderate); *UGT2B11* (moderate); *UGT2B28* (moderate)
hsa-miR-93-3p	*GPX1* (high); *ABCA4* (moderate); *ABCC12* (moderate); *ADH6* (moderate); *CYP4B1* (moderate); *GSTT1* (moderate)

(*continued*)

Supplementary Table S4. (Continued)

MicroRNA	*IPA target (level of confidence for prediction)*
Differentially expressed microRNAs after treatment with RMP	
hsa-let-7a-5p	*UGT8* (experimentally observed); *CYP19A1* (high); *CYP4F2* (high); *FMO2* (high); *FMO4* (high); *KCNJ11* (high); *ABCC10* (moderate); *ABCC5* (moderate); *CHST3* (moderate); *CHST5* (moderate); *CYP46A1* (moderate); *GPX3* (moderate); *GPX7* (moderate); *NR1I2* (moderate); *SLC10A1* (moderate); *SLC10A2* (moderate); *SLC13A3* (moderate); *SLC5A6* (moderate); *SLCO2A1* (moderate); *SLCO5A1* (moderate)
hsa-miR-125b-1-3p	*CAT* (moderate); *HNF4A* (moderate); *PDE3A* (moderate); *TPMT* (moderate)
hsa-miR-1260a	*GSTCD* (high); *KCNJ11* (high); *ADHFE1* (moderate); *CHST11* (moderate); *CYP11B1* (moderate); *CYP11B2* (moderate); *CYP1B1* (moderate); *CYP4F11* (moderate); *CYP4F2* (moderate); *GPX4* (moderate); *SLC27A1* (moderate); *SLC28A3* (moderate); *SLCO4A1* (moderate); *UGT8* (moderate)
hsa-miR-128-3p	*ABCC12* (moderate); *ALDH4A1* (moderate); *CYP1A2* (moderate); *CYP2C19* (moderate); *CYP2C9* (moderate); *CYP39A1* (moderate); *CYP3A4* (moderate); *CYP4F2* (moderate); *GSTM2* (moderate); *PDE3A* (moderate); *PON2* (moderate); *PPARG* (moderate); *SLC5A6* (moderate); *SLC6A6* (moderate); *UGT8* (moderate)
hsa-miR-1291	*ABCC1* (high); *CHST3* (high); *CHST6* (high); *CYB5R3* (high); *CYP1A1* (high); *CYP2A6* (high); *CYP2C18* (high); *GSTZ1* (high); *NR1I3* (high); *PPARD* (high); *SLC5A6* (high); *CHST7* (moderate); *CYP11B2* (moderate); *CYP2S1* (moderate); *CYP39A1* (moderate); *CYP3A5* (moderate); *DHRS13* (moderate); *DHRS7B* (moderate); *GPX3* (moderate); *HNF4A* (moderate); *KCNJ11* (moderate); *SLC16A1* (moderate); *SLC19A1* (moderate); *SLC29A2* (moderate); *SLC6A6* (moderate); *SLC7A5* (moderate)
hsa-miR-139-5p	*ABCA1* (moderate); *PDE3A* (moderate); *SOD1* (moderate)
hsa-miR-195-5p	*GSTM4* (experimentally observed); *CBR3* (high); *GSTCD* (high); *ABCB5* (moderate); *ALDH1A3* (moderate); *ATP7A* (moderate); *CYP2S1* (moderate); *CYP7B1* (moderate); *GPX1* (moderate); *GSTT2/GSTT2B* (moderate); *KCNJ11* (moderate); *NOS1* (moderate); *PDE3B* (moderate); *SLC13A1* (moderate); *SLC22A17* (moderate); *SLC28A1* (moderate); *XDH* (moderate)
hsa-miR-20b-5p	*PPARG* (experimentally observed); *PON2* (high); *ABCC9* (moderate); *ABCG2* (moderate); *ADHFE1* (moderate); *CYP3A5* (moderate); *METAP1* (moderate); *PDE3B* (moderate); *PPARA* (moderate); *SLC29A2* (moderate); *SLC2A4* (moderate)
hsa-miR-212-3p	*ABCG1* (moderate); *CHST9* (moderate); *CYP19A1* (moderate); *CYP4F3* (moderate); *DDO* (moderate); *HSD11B1* (moderate); *SLCO4C1* (moderate); *SOD2* (moderate)
hsa-miR-22-5p	*CYP4Z1* (high); *GSTM2* (high); *GSTT2/GSTT2B* (high); *ABCC1* (moderate); *CYP1A1* (moderate); *GPX5* (moderate); *SLCO1C1* (moderate); *TPMT* (moderate)
hsa-miR-29b-2-5p	*ABCC1* (high); *PDE3A* (high); *ABCA4* (moderate); *CYP2J2* (moderate); *SLC13A3* (moderate); *SLC2A4* (moderate); *SULT2A1* (moderate)
hsa-miR-425-3p	*GSTT1* (moderate); *SLC10A1* (moderate)
hsa-miR-500a	*CYP3A5* (high); *AHR* (moderate); *DHRS2* (moderate)
hsa-miR-577	*ADHFE1* (moderate); *CYP2J2* (moderate); *DHRS9* (moderate)
hsa-miR-597-5p	*ABCB11* (moderate); *ADH1B* (moderate); *CHST6* (moderate); *CYP1B1* (moderate); *SLC5A6* (moderate); *SLCO1A2* (moderate); *SLCO3A1* (moderate)
hsa-miR-625-3p	*CYP3A7* (moderate); *IAPP* (moderate)
hsa-miR-642a-5p	*CYP2A6* (high); *CYP2C18* (moderate); *GPX5* (moderate); *SLC28A1* (moderate)
hsa-miR-876-3p	*CYP3A5* (high); *ALDH1B1* (moderate); *ALDH3B1* (moderate); *CHST6* (moderate); *CYP27B1* (moderate); *CYP2B6* (moderate); *CYP2C18* (moderate); *PPARG* (moderate); *SLC22A12* (moderate); *SLC22A13* (moderate)
hsa-miR-885-5p	*TPMT* (high); *ALDH1B1* (moderate); *UGT2B11* (moderate); *UGT2B28* (moderate)
hsa-miR-93-3p	*GPX1* (high); *ABCA4* (moderate); *ABCC12* (moderate); *ADH6* (moderate); *CYP4B1* (moderate); *GSTT1* (moderate)
hsa-miR-99a-3p	*ALDH1A3* (moderate); *CYP1A1* (moderate); *GSTM5* (moderate); *SLCO4C1* (moderate); *UGT2B4* (moderate)
hsa-miR-99a-5p	*NR1I3* (high)

IPA, Ingenuity Pathway Analysis.
